# AortaExplorer: AI-driven analysis of the aorta in CT images

**DOI:** 10.1007/s11517-026-03535-x

**Published:** 2026-03-16

**Authors:** Rasmus R. Paulsen, Linnea Hjordt Juul, Michael Huy Cuong Pham, Jørgen Tobias Kühl, Klaus Fuglsang Kofoed, Kristine Aavild Sørensen, Josefine Vilsbøll Sundgaard

**Affiliations:** 1 Department of Applied Mathematics and Computer Science, Technical University of Denmark, Kgs. Lyngby, Denmark; 2 Department of Cardiology and Radiology, Copenhagen University Hospital - Rigshospitalet, Copenhagen, Denmark; 3 Department of Clinical Medicine, Faculty of Health and Medical Sciences, University of Copenhagen, Copenhagen, Denmark; 4 Novo Nordisk A/S, Bagsværd, Denmark

**Keywords:** Aorta analysis, Computed tomography, Landmark detection, Segmentation

## Abstract

**Abstract:**

We introduce AortaExplorer, an open-source, fully automated AI-driven framework for end-to-end aortic analysis from computed tomography angiography (CTA) scans. AortaExplorer extracts established biomarkers, such as diameters across anatomical segments, and introduces novel metrics, including aortic tortuosity. Diameter measurements were validated against expert manual readings in more than 10,000 CTA scans from Danish population cohorts, demonstrating high accuracy. The computed descending aortic tortuosity index aligns with trends reported in previous studies, confirming its increase with age. By reducing analysis time from approximately 15 minutes per case to under five minutes, AortaExplorer enables efficient, reproducible assessment of aortic morphology at scale. This tool supports clinical research and large cohort studies by combining state-of-the-art segmentation performance with extensive visualization and quantitative reporting.

**Graphical abstract:**

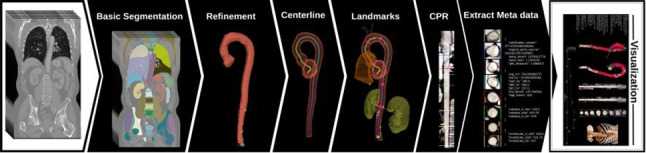

## Introduction

**Fig. 1:**
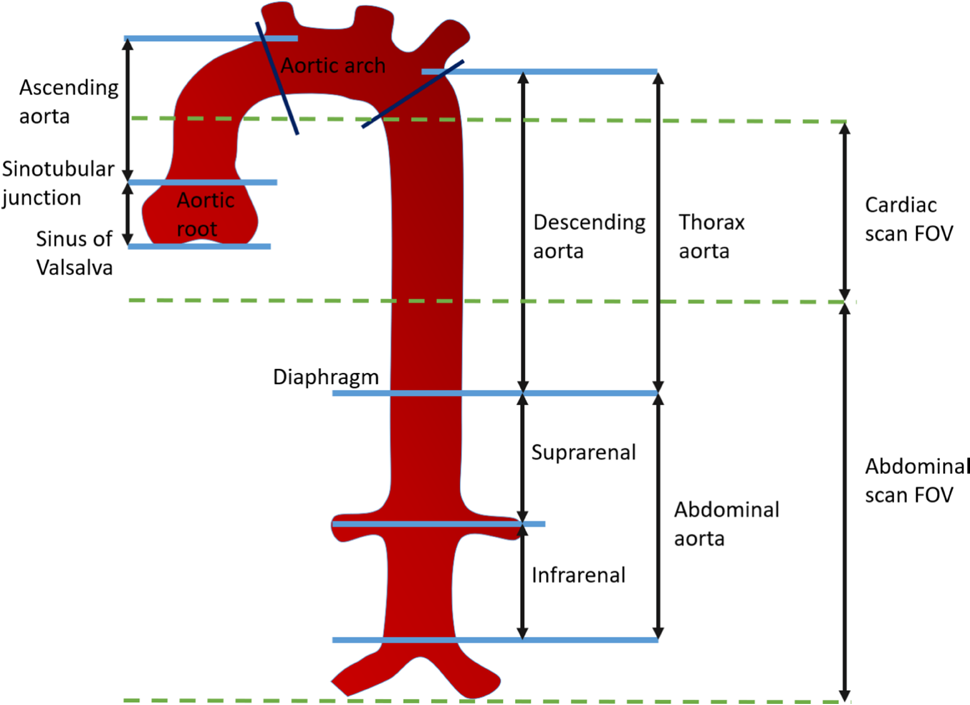
Aortic segments defined by ESC [4], and the field-of-view (FOV) of the cardiac and abdominal CTA scans

The aorta is the main artery in the human body, responsible for carrying oxygenated blood from the left ventricle of the heart and out to the smaller arteries in the systemic circulation. Thus, diseases of the aorta affect the entire systemic circulation. Measurement of aortic diameters is one of the most widely used diagnostics of the aorta [1]. As a biomarker, it is used to estimate the risk of rupture, dissection, and death [2, 3] and is the main risk factor for aortic events [4]. Additional studies have suggested that an enlarged diameter can be a biomarker of other adverse cardiovascular outcomes [5]. Aortic dilation, including aortic aneurysms, has been associated with, for example, cardiovascular disease [5, 6]. Computed Tomography (CT) is the current standard medical imaging modality for the diagnosis, prognosis, and therapy planning of aortic diseases [4]. While Cardiovascular Magnetic Resonance (CMR) can assess the aorta’s shape, size, and tissue without the use of ionizing radiation, CT is used more often because it is more widely available and quicker to perform [4]. Diameter measurements are usually performed manually on 2D CT scans using the inner-to-inner edge method at end-diastole [4]. These intraluminal diameters should be measured at prespecified anatomical landmarks as the maximum distance perpendicular to the centerline. The aorta is typically divided into anatomical segments to define regional measurements, and AortaExplorer follows the definition of ESC [4] as seen in Fig. 1.

Other, more advanced, image-based biomarkers of aortic diseases include, among others, tortuosity. Tortuosity describes the amount of curvature of the blood vessel and is calculated as the ratio between the actual length of the vessel and the geometric length calculated as the Euclidean distance between the end points of the vessel [7]. The tortuosity of the descending aorta has been shown to increase with age [1, 8], due to structural changes in the elastin content of vessel walls, changes in the aortic length [1], and decreased length of the vertebral column [9]. A study in patients with Marfan syndrome found that aortic tortuosity independently predicted aortic dissections [10], and higher tortuosity in the main arteries of the neck has been associated with earlier cardiac surgery, arterial dissection, and death in young adults with connective tissue disorders [11]. However, tortuosity is still a fairly new biomarker and an area of research [1].

In this paper, we present **AortaExplorer**: a fully automatic AI-based framework for the analysis of aortic anatomy from Computed Tomography Angiography (CTA) scans. The purpose of AortaExplorer is to provide standard diagnostics such as aortic diameters in 3D, but also to serve as a research tool that allows exploratory analysis of additional cardiovascular risk predictors such as tortuosity. The framework is built using state-of-the-art building blocks such as TotalSegmentator[12] for basic aorta segmentation and centerline extraction using the Vascular Modeling Toolkit (VMTK), combined with advanced refinement and fine-tuning for this specific application. The main contributions of our work are:

- Publication of an open-source research tool for fully automatic analysis from CTA scan to extracted aortic biomarkers
- Precise segmentation of the true aortic lumen
- Automatic identification of anatomical landmarks for separation into aortic segments
- Automatic computation of regional cross sectional areas and diameters in 3D
- Automatic extraction of aortic tortuosity
- Extensive validation based on manual annotations of aortic diameters in more than 10,000 CTA scans of the Danish general population
- Validation on several open aorta datasets including various aortic diseases
- Advanced visualization of aortic analysis for exploratory and research use

**Fig. 2:**
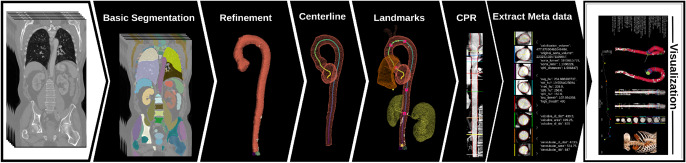
AortaExplorer: Full pipeline from raw CTA scan to final aorta analysis

**AortaExplorer** takes raw contrast-enhanced CTA scans as input and produces a comprehensive visualization of the aorta and its computed statistics. An overview of the pipeline is shown in Fig. 2. **AortaExplorer** is available for research use at https://github.com/RasmusRPaulsen/AortaExplorer/.

## Related work

Automatic methods have previously been developed to assess the diameter of the aorta and detect aortic aneurisms. Adam et al. [13] measured the aortic diameter from the outer wall to the outer wall using a deep learning-based method to segment the aorta. López-Linares et al. [14] presented a 3D Convolutional Neural Network (CNN) for the segmentation of abdominal aortic aneurysms, and Caradu et al. [15] presented a framework for the detection of aortic lumen and infra-renal abdominal aortic aneurysms on CTA scans. The use of post-processing technology in CTA for diagnosing and classifying abdominal aortic aneurysms was also investigated by Yuan et al. [16]. In the AortaSeg24 challenge [17], participants were asked to develop algorithms for automatic segmentation of the aorta in 23 different segments and branches. The challenge was based on a dataset of 100 CTA scans with expert segmentation annotations. The results of the challenge show an impressive segmentation performance of the individual aortic segments, but highlights the lack of datasets and manual annotations in the field [17]. Most deep learning-based aorta segmentation and landmark detection studies are based on a limited number of CT scans (up to 100 scans), and focus only on a smaller part of the aorta, e.g., only the ascending or abdominal aorta [18–23]. Koo et al. [24] developed a multiclass deep learning-based segmentation algorithm on a small part of their available data set, to be able to evaluate the algorithm on scans of 700 healthy individuals and thus provide reference values for the size of the aorta in different segments.

Aortic tortuosity is a relatively new research area; therefore, only a few studies have presented a methodology for automatic assessment of this biomarker. Canals et al. [25] present an analysis of the tortuosity of the supra-aortic region using deep learning for aorta segmentation and a graph neural network for vessel labeling. Moking et al. [26] perform semi-automatic computation of the tortuosity of the common carotid artery and the extracranial internal carotid artery.

Many of the studies presented in this section relies on some kind of deep learning based methods to segment the aorta. The state-of-the-art for segmentation of most major anatomical structures in CT scans was transformed in 2023 with the publication of TotalSegmentator [12]. TotalSegmentator is a robust deep learning-based tool trained on a large set of both CT scans with and without contrast to segment 117 main anatomical structures. TotalSegmentator makes the act of creating baseline segmentations for the aorta and other larger anatomical structures a trivial task, thus widening the landscape of potential research areas within the field of aortic analysis. In this work, we build on top of the best pre-trained models from TotalSegmentator and extend the usability to the more specialized domain of fine-grained aortic analysis.

### Aortic pathologies

**Fig. 3:**
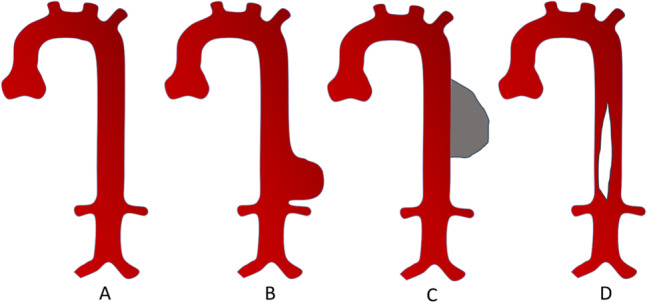
**A**) Normal aorta, **B**) Aorta with a dilation or aneurysm, **C**) Aorta with an aneurysmal sac, **D**) Aorta with a dissection

Aortic dilation, defined as an aortic diameter exceeding two standard deviations above the expected mean diameter, is the second most common aortic pathology [4]. A localized dilation exceeding 150% of the predicted diameter constitutes an aortic aneurysm, which typically appears as a contrast-filled segment on CT images. In some cases, a minor leak in the aortic wall leads to an aneurysmic sac, where blood accumulates and coagulates; this structure is visible on CT but does not contain contrast-enhanced blood. An aortic dissection occurs when the intimal layer is breached, allowing blood to separate the layers of the wall. Most aneurysms are clinically silent–particularly abdominal aneurysms, where two-thirds of cases are asymptomatic [4]. The primary symptomatic event is rupture, which is often fatal even with immediate intervention. Early management through medication or lifestyle modification is, therefore, critical to prevent rupture [2, 27, 28]. Figure 3 provides an overview of these aortic pathologies, while Fig. 15 illustrates representative cases from the dataset. Although AortaExplorer is not intended for diagnostic use, severe cases appear in the external evaluation datasets.

## Data

The Copenhagen General Population Study (CGPS) is an ongoing prospective cohort study in Denmark. Since 2010, participants of at least 40 years of age with normal kidney function have been offered the opportunity to undergo a cardiac CTA research examination at Copenhagen University Hospital in Denmark. The scan protocol was extended with an abdominal CTA scan in 2014. Finally, a novel scan protocol was used to acquire the full aorta. The study protocol was approved by both a regional ethics committee (H-KF-01-144/01) and a local institutional review board. Participant consent is obtained for all cases analyzed. CTA image acquisition was performed using a 320-slice multi-detector computed tomography scanner. Abdominal and full aorta CTA was reconstructed with 0.5 mm slice thickness and increments of 0.30 mm, while cardiac CTA was reconstructed with 0.5 mm slice thickness and increments of 0.25 mm. Cardiac CTA was scanned using ECG gating in the 75% phase of the R-R interval. A complete description of the scan protocol is found in [29].

To evaluate the performance of AortaExplorer, examinations using three different scan protocols are used:

- Cardiac CTA: $N = 11,418$ with manual measurements of aortic diameters.
- Abdominal CTA: $N = 7,627$ with manual measurements of aortic diameters. These are a subgroup of participants with cardiac CTA.
- Full aortic CTA: $N = 1,144$ without manual measurements.

**Fig. 4:**
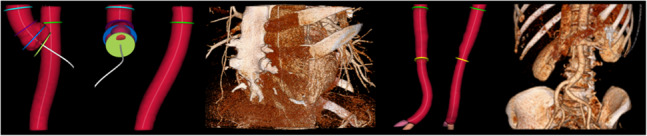
FOV of the various CTA scans. *Left: *Cardiac CTA. *Right:* Abdominal CTA. The aorta surfaces are shown from two different view points

**Fig. 5:**
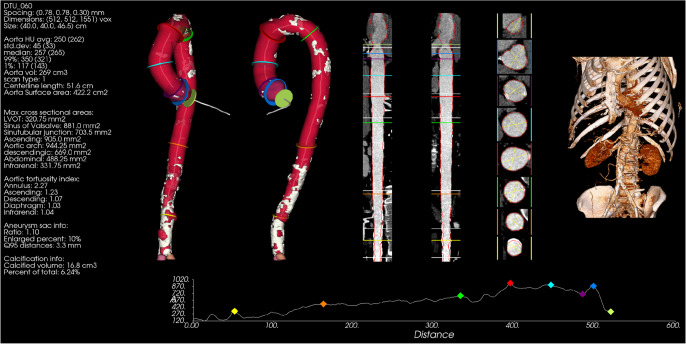
A visualization of the analysis of an entire aorta using AortaExplorer

The field-of-view (FOV) is different for cardiac and abdominal CTA scans and is shown in Fig. 1, while Fig. 4 shows examples of cardiac and abdominal CTA scans. A complete aortic CTA scan can be seen in Fig. 5, which shows the full analysis output from AortaExplorer.

Manual measurements of aortic diameters were performed by an experienced medical reader at six anatomical locations: annulus, sinotubular junction, sinus of Valsalva, ascending aorta, descending aorta, and infrarenal segment. Diameters at the annulus, sinotubular junction, sinus of Valsalva, ascending, and descending segments were obtained from cardiac CTA scans, while the infrarenal diameter was measured on abdominal CTA scans. Measurements were taken in regions without aneurysms; if an aneurysm was present, its diameter was recorded separately.

### External datasets for validation

AortaExplorer robustness is evaluated by processing a set of open-source datasets of the aorta:

- The **AortaSeg24** dataset [17] contains 100 CTA images with associated ground truth segmentations of the aorta and its segments. Visual inspection of the images shows that most patients have a type B aortic dissection, which is a tear in the inner layer of the aorta that starts in the descending aorta (see Fig. 3).
- The **Aortic Vessel Tree (AVT)** CTA dataset [30] includes 56 CTA scans, primarily from healthy aortas, covering the arch with branches and the abdominal aorta with iliac arteries. Each scan is accompanied by segmentation masks of the aorta and its branches.
- The **CIS-UNet** dataset [18] contains 59 CTA scans of the aorta. The data set also includes several ground truth masks, including the aorta and its branches. It seems to have a partial overlap with the AortaSeg24 data set [17] and also mostly contains cases with aortic dissections [31].

## Methods

Each step seen in the analysis pipeline in Fig. 2 is explained below.

### Basic segmentation and refinement

The baseline segmentation of the aorta is computed using TotalSegmentator v.1.5 (TS) [12] pre-trained total model. We also use the left ventricular (LV) and right ventricular (RV) blood pools computed using the heartchambers_highres pre-trained model. The TS segmentation of the aorta includes the full anatomical structure of the aorta, including the aortic wall, potential aneurysmal sacs, calcifications, and dilations.

To allow for comparison with the measurement protocol in clinical practice, an accurate segmentation of the pure lumen (contrast-enhanced blood) is needed. Therefore, the TS segmentations need to be sub-segmented into a pure lumen and a calcification segmentation. An AortaExplorer result can be seen in Fig. 5. The original TS segmentation includes blood (lumen), calcifications, and the aortic wall.

A skeleton is generated from the TS segmentation using morphological skeletonization [32] and dilated by 1 mm to represent pure lumen voxels. Their Hounsfield unit (HU) values are sampled to compute the mean ($\mu _\text {HU}$) and standard deviation ($\sigma _\text {HU}$). Following [33], the lumen is estimated as connected voxels within the HU range $[\mu _\text {HU} - 5\sigma _\text {HU}, \mu _\text {HU} + 3\sigma _\text {HU}]$, excluding low HU values from the aortic wall and high HU values likely corresponding to calcifications. Morphological opening and closing are applied as post-processing to remove holes and outliers. An ablation study is carried out in section 5.3 to assess the effect of changing the thresholding parameters.

### Centerline extraction

The aorta centerline is computed using the vascular modeling toolkit (VMTK) [34] which, based on a surface representation of the aorta and a start and an end point, provides a centerline that follows the centers of the maximally inscribed spheres in the aorta. The surface of the aorta is computed using a discrete marching cubes algorithm on the segmentation of the lumen of the aorta [35]. For full-aorta and abdominal-aorta scans, the start point is the iliac bifurcation identified from the TS aorta and iliac segmentations. The abdominal aorta end point is where the aorta leaves the upper scan range, while the full-aorta end point is on the LV at the distal aortic connection. For cardiac scans, the descending aorta uses the scan entry and exit points, whereas the ascending aorta starts where it leaves the scan and ends on the LV at the distal aortic connection.

**Fig. 6:**
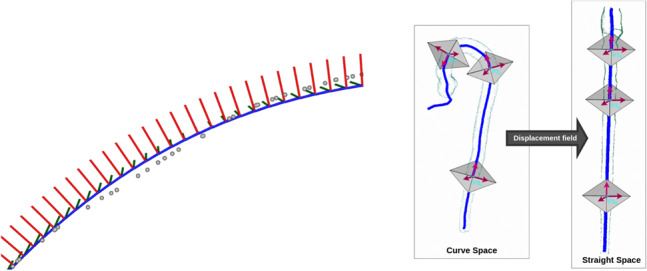
*Left:* Spline approximation and its local coordinate system. Original center line points in gray. *Right:* Curved Planar Reformatting: Each point on the centerline acts as origin in a local coordinate system, that is used to sample slices that are stacked in a straight volume

The centerline found by VMTK is approximated by a smoothing spline [36] to remove minor fluctuations and to robustly compute the local tangent. From the tangent and a defined up-vector, the normal and binormal can also be computed. The tangent, normal, and binormal form a local coordinate frame. This is also called a Frenet-Serret frame. A regularization step avoids sharp changes in the frames along the curve as described in [37]. An inflection point of a curve is where a change in the direction of curvature occurs. This will induce a flipping of the curve normal. Following [37], the dot product between the normal of the previous position on the curve and the normal at the current position is computed and if negative, the normal in the current position is flipped. This ensures a stable propagation of frames along the curve without flipping normals. See Fig. 6 (left) for an example of a spline approximation of a centerline (gray points), with the local frames shown. The local frames are used in the following curved planar reformatting.

### Curved planar reformatting

Curved Planar Reformatting (CPR) exploits the tubular structure of the aorta, resampling it in order to produce a straightened vessel [38]. CPR maps from the original scanning space to a new space, where the vessel is straight. The main principle of this mapping is illustrated in Fig. 6 (right). Each point on the curved centerline defines the origin in a local coordinate system defined by the regularized Frenet-Serret frames computed on the centerline, such that the xy-axes span a plane orthogonal to the curve. A user defined sampling distance can be used to sample slices along the curve and stacking them into the final straight volume. An example of a straightened volume can be seen in Fig. 5. The lumen segmentation is also straightened and used to calculate cross-sectional areas and diameters.

### Identification of aortic landmarks

**Fig. 7:**
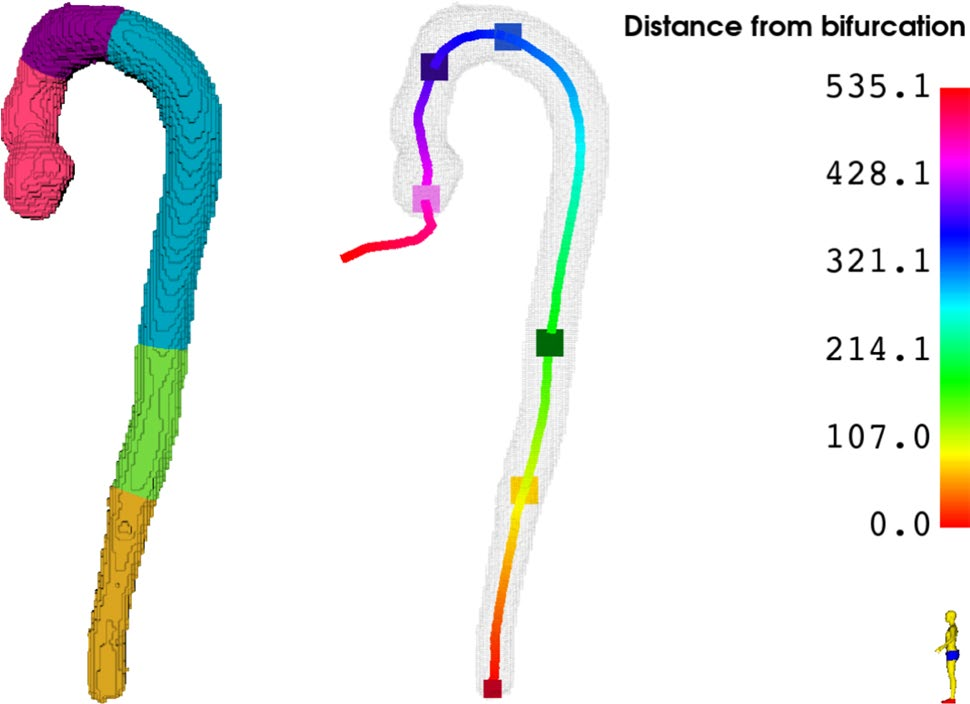
Computation of centerline landmarks. *Left:* The aorta divided into segments - Ascending (pink), aortic arch (purple), descending (turquoise), suprarenal (green), and infrarenal (orange). *Right:* Centerline landmarks - Ventricularaortic (rosa), proximal aortic arch landmark (dark purple), distal aortic arch landmark (blue), diaphragm (green), renal (yellow) and aortic endpoint at the bifurcation (dark red)

We follow the 2024 ESC Guidelines for the management of peripheral arterial and aortic diseases [4] to divide the aorta into anatomical segments and the identification of anatomical landmarks, as seen in Figs. 1 and 7. Our landmark detection method works by first locating anatomical landmarks based on the segmentations and then projecting these on the centerline. This divides the centerline into the corresponding anatomical segments. For a scan that contains the entire aorta, five points on the centerline are chosen as landmarks, dividing the aorta into anatomical segments; ascending, arch, descending, suprarenal and infrarenal, as shown in Fig. 7. The number of available landmarks depends on the scan type, as the field-of-view (FOV) varies between scans.

The identification of landmarks begins at the ventricular–aortic junction, marked pink in Fig. 7 (right), and proceeds along the centerline to the bifurcation, shown in dark red. The ventricular–aortic landmark represents the boundary between the left ventricle (LV) and the aorta. To compute its position, the LV segmentation from TS and the refined aortic segmentation are dilated, the center of mass of their overlap is calculated, and the nearest centerline point is assigned as the landmark.

**Fig. 8:**
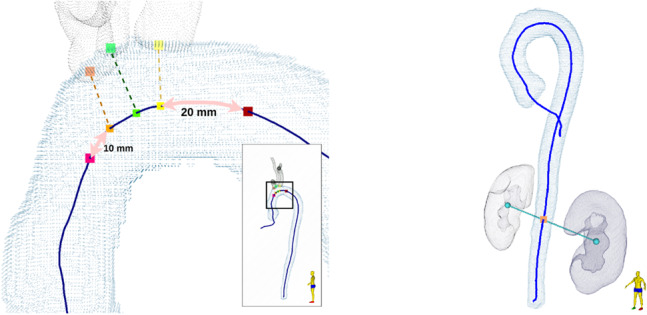
Computation of landmarks. *Left*: Computation of the proximal (pink) and distal (red) aortic arch landmarks using landmarks for the brachiocephalic trunk (orange), left common carotid (green), and subclavian arteries (yellow). *Right:* Computation of renal landmark using the center of mass of kidney segmentations

The landmarks on the aortic arch include the proximal aortic arch landmark at the origin of TS segmentation of the brachiocephalic trunk, and the distal aortic arch landmark that is approximately 2 cm distal to the TS segmentation of the left subclavian artery. The landmarks of the three arteries that originate in the aortic arch are determined in a similar fashion, using TS segmentations for the three arteries, applying dilation with a structuring element with a radius of 5 mm in both the artery segmentation and the refined segmentation of the aorta, and calculating the center of mass of the overlap. From these, the nearest corresponding points on the centerline are computed and the final aortic landmarks are calculated as shown in Fig. 8.

**Fig. 9:**
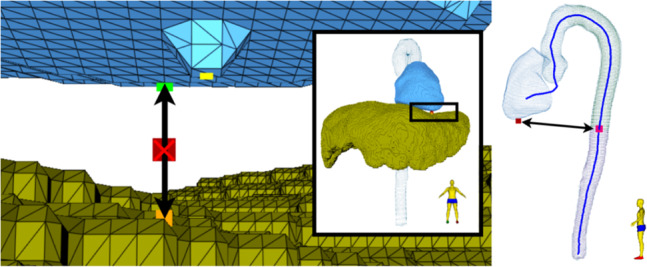
Computation of diaphragm landmarks. *Left:* Computation of diaphragm placement (red) via. the left ventricle (blue) and the liver (yellow). *Right:* Landmark of diaphragm on the aortic centerline, computed as the centerline point (pink) with the shortest distance to the estimated diaphragm placement (red)

The descending aorta extends from the distal arch landmark to the diaphragm. The approximate location of the diaphragm is estimated using the TS segmentations of the right ventricle (RV), located above, and the liver, located below, as shown in Fig. 9(left). Starting from the lowest RV point, the shortest RV–liver distance is iteratively computed, resulting in two points. Their midpoint defines the diaphragm, and the nearest centerline point becomes the diaphragm landmark (Fig. 9(right)).

The abdominal aorta is divided into suprarenal and infrarenal segments by the renal landmark, ideally placed at the origin of the renal arteries. However, since this intersection is often poorly visualized due to limited contrast, the landmark is instead computed from kidney TS segmentations: the line connecting both kidney centers of mass is drawn, and the closest centerline point is selected as the renal landmark (Fig. 8(right)).

**Fig. 10:**
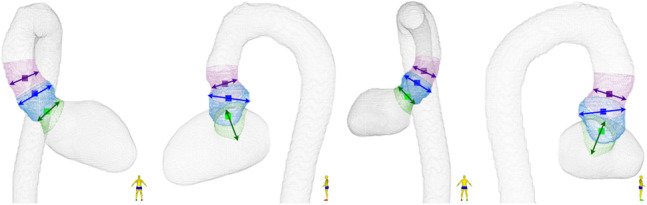
Landmarks diameter measurements in the aortic root and annulus. The areas are highlighted for the sinuses of Valsalva (blue), the area examined for the presence of the sinotubular junction (purple) and annulus (green), with the computed landmarks shown in the corresponding colors

In addition to these five segmental landmarks, three landmarks characterize the aortic root, as seen in Fig. 10. The sinus of Valsalva is located within 20 mm distal to the ventricular–aortic landmark; the point with the largest cross-sectional area within this segment is chosen. The sinotubular junction is identified as the smallest cross-sectional area between 5 and 25 mm beyond the sinus landmark. Finally, the aortic annulus is defined as the minimum cross-sectional area within 10 mm proximal to the ventricular–aortic landmark along the combined LV–aorta centerline.

### Extraction of aortic biomarkers

AortaExplorer automatically extracts the following image-based aortic biomarkers: diameter measurements and cross-sectional areas at defined landmarks and aortic tortuosity.

The cross-sectional area can be extracted from the straightened segmentation of the aortic lumen. Diameter measurements are performed where the cross-sectional area is the largest (smallest for the sinotubular junction and annulus) for each of the anatomical segments shown in Fig. 7(left), as well as the landmarks for the sinus of Valsalva, sinotubular junction, and annulus. The maximal diameter is defined as the maximally long line that goes through the center of mass of the cross section and hits the boundaries of the cross section.

Blood vessels have characteristic curved shapes, and tortuosity of vessels is most commonly defined as the ratio between the actual length of a vessel and the geometric length calculated as the Euclidean distance between the end points of the vessel [7]. AortaExplorer computes two tortuosity measurements for the aorta: descending aortic tortuosity and full aortic tortuosity. The descending aortic tortuosity is calculated as the ratio between the geometric distance between the distal aortic arch and the diaphragm landmark and the actual distance following the center line between the two points. Similarly, the full aortic tortuosity is computed using the geometric and center line distances between the iliac bifurcation and the ventricularaortic landmark.

### Visualization

AortaExplorer is intended as a research tool for the exploratory analysis of the aorta. It is therefore crucial to provide high-quality visualizations of the results for human visual validation and as a learning tool. For each case, a visualization as seen in Fig. 5 is rendered as a PNG file. The visualization includes from left to right: metadata and extracted biomarkers; surface rendering of the aorta segmentation in two different views with indication of separation into aortic segments and excluded calcifications; CPR representation of the aorta in two planes with segmentation outline and diameter measurement locations drawn on top of the CTA scans; cross sectional CTA slices of the aorta at diameter measurement locations; volume rendering of the body from the CTA scan. At the bottom of the visualization is a plot of the cross-sectional area of the aorta along the centerline with markings where diameters are measured. All visualizations are performed using a combination of SimpleITK [39] and the Visualization Toolkit (VTK) [40]. Surface rendering is done by first transforming segmentations into triangulated surfaces using marching cubes [35]. The volume rendered body is done by a GPU accelerated VTK renderer using a custom look-up table for tissue colors and opacities.

### Detection of failure cases

AortaExplorer implements multiple safeguards to handle incomplete scans and acquisition errors. The first step determines the scan’s field-of-view (Fig. 1) by assessing whether the aorta appears as a single structure or two separate segments, as in cardiac scans. A landmark-based heuristic then classifies the scan as complete, abdominal, or a two-part cardiac scan. Additional checks identify partial coverage, such as missing descending aorta. Subsequent analysis is restricted to well-defined cases. After lumen segmentation, the surface-to-volume ratio is computed. High ratios indicate a rough or porous lumen surface, typically reflecting non-contrast imaging or acquisition artifacts. This metric successfully flagged mislabeled non-contrast scans during development. For each cross-sectional slice, the aspect ratio of the best-fitting ellipse is also evaluated; if it exceeds a predefined threshold, the diameter estimate is discarded, and a warning is raised.

### Statistical analysis

Aortic diameter measurements were evaluated against manual annotations using multiple metrics: mean absolute error (MAE), to quantify average deviation; mean absolute percentage error (MAPE), to show the estimation error compared to true aorta diameters; bias, also called mean error, to assess over- or underestimation. Furthermore, estimated diameters and manual measurements were evaluated as an inter-observer study by computing the Pearson correlation coefficient between the two variables and the coefficient of variation. The coefficient of variation is estimated as the relative bias by computing the mean of the difference between automated and manual estimates over the average of the two estimates as in [29]. Bland-Altman analysis was performed to evaluate inter-observer (AI versus manual) agreement. All statistical tests for tortuosity were performed using the Python library *scipy*. Normality was tested using the Shapiro Wilk test. Comparison of mean values in two groups was performed using the *t*-test for normally distributed data and using the Mann-Whitney U-test for non-normally distributed data. Linear regression analysis was used to assess the effect of age on tortuosity. A significance level of $\alpha = 0.05$ was used for all analyzes.

## Results

The aim of AortaExplorer is to enable AI-driven image reading for aortic analysis. The results produced by the pipeline contain a mixture of visualizations along with the computed statistics of the aorta, including the measurement of diameters at several landmarks and the tortuosity index. An example of an AortaExplorer analysis can be seen in Fig. 5.

Experiments were conducted on a desktop system with 128 GB RAM, an AMD Ryzen 7 7800X3D CPU, and an NVIDIA GeForce RTX 4090 GPU (12 GB VRAM). Total analysis time, including TotalSegmentator, was approximately five minutes per scan. Peak GPU memory usage was 8 GB during segmentation, and peak CPU memory usage was about 10 GB per process. The implementation is not optimized for speed, and runtime could thus be reduced further. AortaExplorer supports NIFTI, NRRD, and DICOM formats, enables batch processing of large datasets, and uses a multi-processing framework that leverages all available CPU cores. The software has been tested in a Danish hospital and runs on consumer-grade hardware. Additional details and examples are available on GitHub^1^.

**Table 1:** Automatic measurement (TS and AortaExplorer) compared to manual measurement of aortic diameters

Diameter	Metric	Raw TS seg.	Refined seg.
Annulus	Samples (N)	-	11,007
	MAE	-	1.52 mm
	MAPE	-	5.70$\%$
	Bias	-	-1.08 mm
	Pearson	-	0.85
	CV	-	3.96$\%$
	Out of error range	-	4.45$\%$
Sinus of Valsalva	Samples (N)	11,004	11,004
	MAE	4.10 mm	2.32 mm
	MAPE	12.37$\%$	6.99$\%$
	Bias	-4.08 mm	-2.10 mm
	Pearson	0.90	0.90
	CV	11.39$\%$	6.01$\%$
	Out of error range	4.14$\%$	4.03$\%$
Sinotubular junction	Samples (N)	10,963	10,963
	MAE	3.49 mm	1.06 mm
	MAPE	12.22$\%$	3.68$\%$
	Bias	-3.48 mm	-0.85 mm
	Pearson	0.94	0.94
	CV	11.26$\%$	2.88$\%$
	Out of error range	2.75$\%$	2.62$\%$
Ascending	Samples (N)	10,958	10,958
	MAE	3.57 mm	1.54 mm
	MAPE	11.29$\%$	4.85$\%$
	Bias	-3.56 mm	-1.41 mm
	Pearson	0.95	0.95
	CV	10.48$\%$	4.28$\%$
	Out of error range	4.26$\%$	4.02$\%$
Descending	Samples (N)	11,003	11,003
	MAE	2.43 mm	0.97 mm
	MAPE	10.38$\%$	4.11$\%$
	Bias	-2.40 mm	-0.33 mm
	Pearson	0.90	0.89
	CV	9.59$\%$	1.39$\%$
	Out of error range	3.99$\%$	4.32$\%$
Infrarenal	Samples (N)	7,275	7,275
	MAE	3.11 mm	1.77 mm
	MAPE	16.58$\%$	9.40$\%$
	Bias	-3.09 mm	-1.53 mm
	Pearson	0.85	0.83
	CV	14.97$\%$	7.70$\%$
	Out of error range	5.25$\%$	5.20$\%$

MAE is mean absolute error, MAPE is mean absolute percentage error, CV is coefficient of variation

**Fig. 11:**
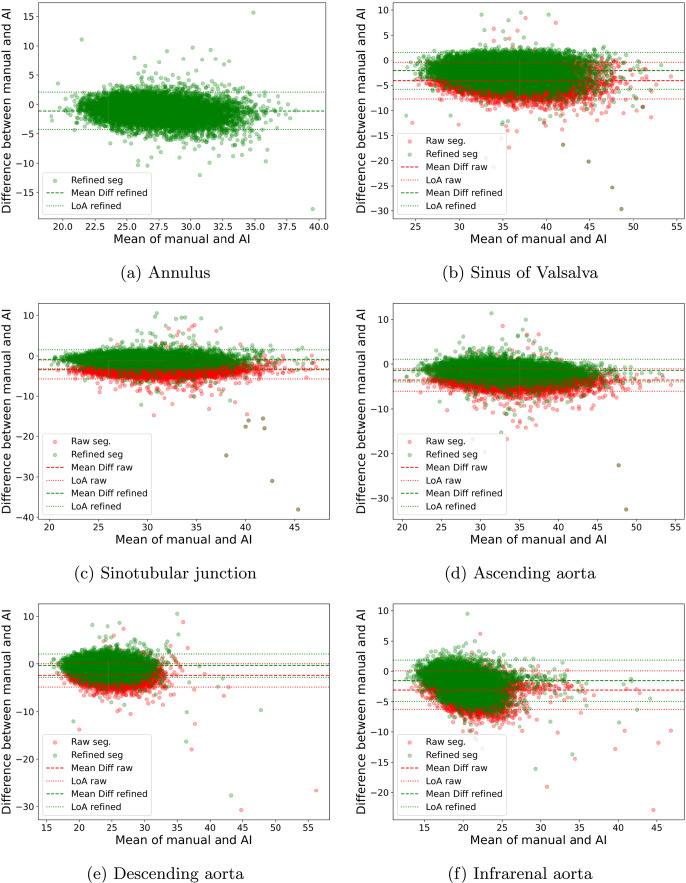
The Bland-Altman plots showing the manual measurements of the aortic diameter versus AortaExplorer (green) and TS segmentations (red) at different anatomical segments. Annulus does not have a TS segmentation measurement

### Diameter measurements

The Bland-Altman plots in Fig. 11 present the inter-observer analysis comparing the manual and automatically measured diameter measurements. The plots show results for both the raw TS segmentations and the refined segmentations (AortaExplorer output), as described in Section 4.1. Table 1 shows the performance of the AortaExplorer (refined) and TS segmentations (raw) compared to manual measurements. The annulus diameter measurement cannot be measured on the raw TS segmentations, and thus only the AortaExplorer results are shown. The results show that only a few samples (min: 2.62%, max: 5.20%) lie outside the $\pm 1.96$ standard deviation confidence interval for the difference in aortic diameter. AortaExplorer reports the maximum diameter within each segment (except at the sinotubular junction). In contrast, manual measurements rely on the reader’s judgment regarding aneurysm presence and whether to measure outside the dilated region. These decisions introduce variability and represent a major source of outliers observed in the Bland–Altman plots. The Bland-Altman plots show that there is a bias in our automatic estimates of aortic diameters, which is also seen in Table 1. All diameters are slightly overestimated; however, the results show that our refinement of the raw TS segmentations improves this issue by decreasing the bias for all diameter measurements. Performance metrics MAE and MAPE also show that the diameter estimation error is smaller for refined AortaExplorer segmentations. There are still some extreme outliers clearly visible in the lower right corners of the Bland-Altmann plots in Fig. 11, and these are typically disagreements between manual reading and automatic measurement in aortas with dilations. An analysis of failure cases can be found in section 5.4. The goal of AortaExplorer is to provide clinical researchers with visualization of the results for a sanity check of the biomarkers generated.

The coefficient of variation (CV) for diameter measurements are slightly higher than the inter-observer variability between two independent manual readers, as reported by Pham et al. [29]: 5.3%, 2.9%, 2.8%, 5.6%, and 5.1% for sinus of Valsalva, sinotubular junction, ascending, descending, and infrarenal diameters, respectively, but still within an acceptable range for being used as a clinical research tool (standard acceptable CV level is 10%).

### Tortuosity

**Fig. 12:**
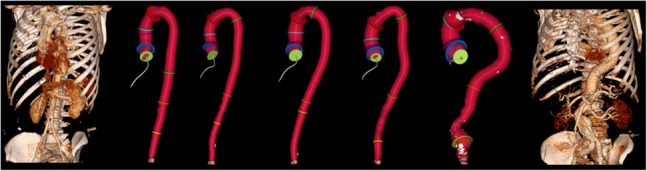
Five examples of increasing descending tortuosity index. From left to right: 1.014, 1.069, 1.099, 1.140, 1.444. Volume renderings of the two extremes are also shown. The scan to the right also indicates a severe infrarenal dilation of the aorta

**Fig. 13:**
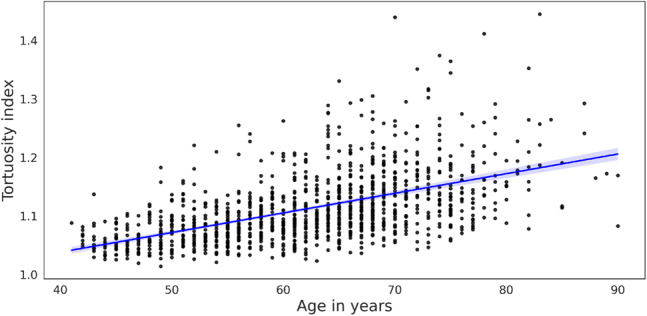
Tortuosity index of descending aorta in relation to age. Linear regression line in blue with 95% confidence interval for the regression estimate as shaded blue background

Examples of aorta scans with an increasing tortuosity index can be seen in Fig. 12. The tortuosity index is not manually assessed on CT scans, so a direct AI versus manual measurements comparison cannot be performed. However, the distribution of the tortuosity index measurements of our cohort of 1,174 full aortic CTA scans can be investigated and compared with previous related work by Belvroy et al. [8]. The previous study shows that the tortuosity of the descending aorta increases with age and that the tortuosity is significantly higher for people over 65 years of age compared to those under 65 years of age (1.05 versus 1.14, respectively; $p < 0.001$) [8]. Our analysis of tortuosity in relation to age is shown in Fig. 13, and the difference between the two age groups (above and below 65 years) is analyzed in Table 2. Our results show that both descending and full aortic tortuosity increases with age and is significantly higher for participants over 65 years of age compared to younger participants. The linear regression plotted in Fig. 13 shows a moderate correlation between tortuosity and age ($r = 0.54, p<0.001$). Table 2 also shows that the geometric length of the aorta decreases with age, while the centerline length of the aorta increases, both contributing to the higher tortuosity index at older ages. All of our tortuosity results align with Belroy et al. [8], and thus we conclude that our automatic computation of tortuosity has been validated.

**Table 2:** Comparing tortuosity measurements of participants above and below 65 years old

	<65y	$\ge$65y	p-value
Samples (N)	700	444	
Descending TI	1.09 ± 0.046	1.15 ± 0.064	$<0.001^*$
Full aortic TI	2.04 ± 0.14	2.20 ± 0.16	$<0.001^*$
Geometric length	242.25 ± 19.87	232.24 ± 19.43	$<0.001^*$
Centerline length	492.77 ± 36.45	508.84 ± 38.22	$<0.001^*$
[8] Samples (N)	100	100	
[8] Descending TI	1.05 ± 0.024	1.14 ± 0.078	$<0.001^*$

Our study results are presented above the midline, whereas below the midline are the results from Belroy et al. [8]. TI = Tortuosity Index. Mann-Whitney U-test was used for both TI, standard t-test is used for both length measurements. * indicates significance at $\alpha=0.05$

### Calcifications

To identify which of the voxels removed during the segmentation refinement that can be considered calcifications, we use an adaptive HU threshold defined as $\max (v_\text {minhu}, \mu _\text {HU} + \alpha \sigma _\text {HU})$, where $v_\text {minhu}$ is a minimum HU value (default: 400) and $\alpha$ is a weighting factor (default: 3). To assess the robustness of the calcification extraction we conduct an ablation study on the thresholding parameters. In Table 3 it can be seen how varying the parameters $v_\text {minhu}$ and $\alpha$ influences the estimate of the calcification ratios in these scans. Since the calcification threshold also influences the lumen estimate, the lumen estimates are compared at the infrarenal section using the manually estimated maximum diameters. Default parameters ($\alpha =3$, $v_\text {minhu}=400$) achieved a balance between low MAE, MAPE, bias, and CV while maintaining consistent calcification estimates. When $v_\text {minhu}<400$ and $\alpha <3$, the algorithm will not be able to compute the lumen accurately, whereas higher thresholds ($\alpha>3$, $v_\text {minhu}>600$) risked including calcified regions in the lumen. Diameter estimates remained robust provided $\alpha \ge 1$ and $v_\text {minhu} \ge 400$.

**Table 3:** Diameter and calcification estimation when varying parameters $\alpha$ and $v_\text {minhu}$: The mean and standard deviation (std) of the estimated calcification ratios, estimated infrarenal maximum diameter compared with the manual reader estimate of the diameter (MAE, MAPE, bias, CV), and the number of failed cases

$\alpha$	$v_\text {minhu}$	Mean	Std	MAE (mm)	MAPE (%)	Bias (mm)	CV (%)	*N* failed
1	200	15.06	8.04	3.00	16.29	1.30	6.62	4
1	300	10.83	10.35	3.03	16.51	1.36	6.91	4
1	400	5.14	10.21	2.96	16.04	1.43	7.21	2
1	600	0.73	1.98	3.01	16.32	1.54	7.77	0
1	800	0.23	0.65	2.97	16.13	1.59	8.02	0
3	200	1.27	2.48	3.02	16.29	1.46	7.39	0
3	300	1.24	2.47	3.02	16.29	1.46	7.39	0
**3**	**400**	**1.10**	**2.47**	**3.03**	**16.37**	**1.48**	**7.45**	**0**
3	600	0.65	1.78	3.01	16.32	1.54	7.77	0
3	800	0.23	0.65	2.97	16.13	1.59	8.02	0
5	200	0.79	1.70	3.00	16.21	1.52	7.69	0
5	300	0.79	1.70	3.00	16.21	1.52	7.69	0
5	400	0.77	1.68	3.00	16.23	1.53	7.71	0
5	600	0.54	1.40	3.03	16.38	1.55	7.82	0
5	800	0.23	0.65	2.97	16.13	1.59	8.02	0

Bold marks the optimal parameters

**Fig. 14:**
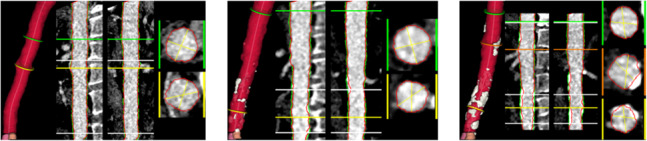
Three scans with increasing calcification ratios. From left to right 0%, 3%, and 11%

Figure 14 shows examples of cases with varying amounts of calcification in the abdominal aorta, as estimated by AortaExplorer. Here, it is evident that AortaExplorer excludes calcium deposits from the original segmentation.

### Analysis of failure cases and outliers

**Fig. 15:**
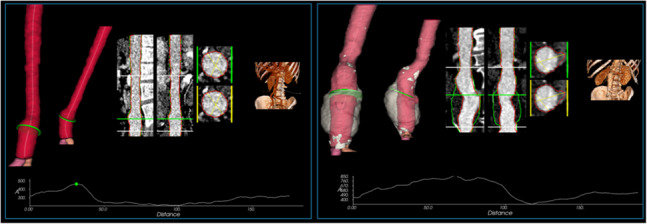
Outlier measurements for infrarenal maximum diameters. *Left:* Manual measurement = 16.1 mm, AortaExplorer = 27.4 mm and TotalSegmentator = 28.8 mm. *Right:* Manual measurement = 33.1 mm, AortaExplorer = 38.7 mm and TotalSegmentator (green curve and transparent surface) = 56.0 mm

As described in Section 4.7, AortaExplorer applies multiple error checks. To quantify failure rates and outliers, sources of error and reasons for scan exclusion were analyzed. 7,627 abdominal scans were available for analysis, and AortaExplorer failed in 127 cases, resulting in a failure rate of 127/7,627 = 1.67% . Manual inspection showed that the vast majority of cases were due to incorrect contrast labeling during CT acquisition or an incomplete field-of-view, where the aorta was not visible. In total, 7,471 scans were successfully processed, and 7,275 of these cases had manual measurements. For cardiac scans, 11,418 cases were available, and 145 scans failed for similar reasons (failure rate = 1.45%). Table 1 summarizes the number of scans with successfully measured diameters. For non-contrast cases or where the aorta is not visible, no aortic measurements are reported. For cases with a partially visible aorta, AortaExplorer reports a partial set of measurements and warns about missing values. Figure 15 illustrates typical outliers. In the left example, both AortaExplorer and TotalSegmentator measured the diameter within an aneurysm (defined in Fig. 3), whereas the manual measurement was taken outside the aneurysm, which was not reported by the reader. Visual inspection indicates this as the most frequent cause of discrepancies. In the right example, a large aneurysmal sac is present; both AortaExplorer and the manual reader measured the contrast-enhanced lumen, while TotalSegmentator included the sac. Since AortaExplorer is designed to report diameters of the flowing blood lumen, this behavior is consistent with this objective.

### Validation of AortaExplorer on public data

To test the robustness of AortaExplorer, the performance has been tested on a set of open-access datasets. Detailed information on how to run AortaExplorer on these datasets can be found on the Github page^2^, including more examples and discussions of the results. A brief overview of the validation results can be seen in Table 4.

**Table 4:** Overview of validation of AortaExplorer on public data

Dataset	Diseases	$N_{fail}/N_{cases}$	Dice: avg.[min, max]	Failure reasons
AortaSeg24 [17]	Type B dissections	1/100 (1%)	0.84 [0.61, 0.93]	TS seg. error
AVT [30]	Healthy and dissections	7/56 (12.5%)	0.87 [0.71, 0.95]	Absent contrast Image corruption TS seg. error
CIS-Unet [18]	Healthy and dissections	0/59 (0%)	0.85 [0.65, 0.94]	None

#### AortaSeg24

**Fig. 16:**
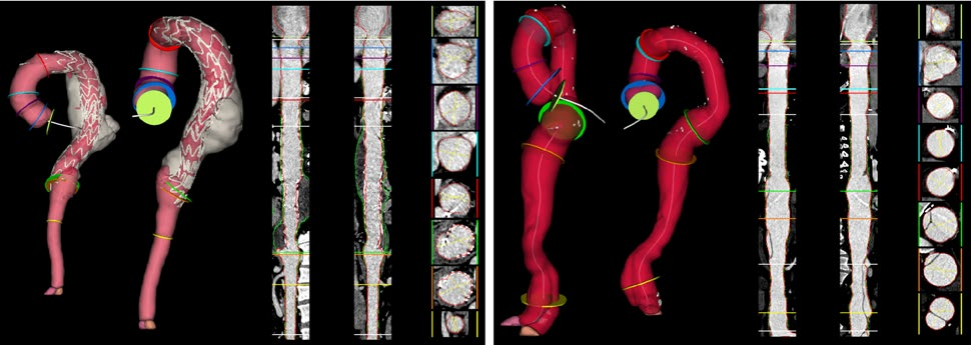
AortaExplorer result on a cases from the AortaSeg24 dataset. *Left:* Case 011, the red 3D aorta is the pure lumen found by AortaExplorer and the transparent surface is the aorta segmentation provided by TotalSegmentator. *Right:* Case 090, patient with aortic type B dissections

**Fig. 17:**
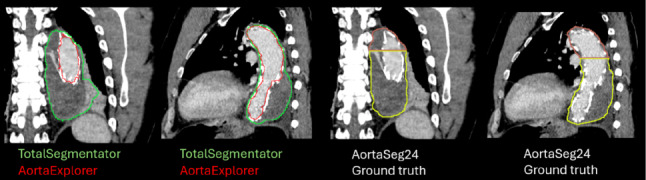
Comparison of segmentation: *Left:* AortaExplorer (red), TotalSegmentator (green). *Right:* AortaSeg24 provided groundtruth of two different aortic segments (brown and yellow)

Although AortaExplorer is not intended as a diagnostic tool for aortic dissection, it successfully processed 99 of 100 AortaSeg24 cases; the single failure occurred because TotalSegmentator did not segment the entire aorta. Fig. 16 (left) shows an example where the patient appears to have undergone thoracic endovascular aortic repair (TEVAR), with a metallic stent visible as a grid-like pattern on the lumen surface. A comparison of segmentations from AortaExplorer, TotalSegmentator, and the ground truth is presented in Fig. 17. It can be seen that the ground truth segmentations and the TotalSegmentator segmentations include both the true lumen and the large aortic aneurysmal sac (see Fig. 3 for reference). Since the goal of AortaExplorer is the segmentation of the true lumen (contrast filled blood), the segmentations are not directly comparable. Visual inspection shows that AortaExplorer correctly segments the lumen. Another example can be seen in Fig. 16 (right), where the patient has aortic dissections indicated by the darker lines inside the cross sectional images. Several aneurysms can also be identified from the local maxima in the diameter plot. Across all 99 successful cases, visual inspection confirmed type B dissections, suggesting that AortaExplorer could serve as an initial visual screening tool in such scenarios.

Dice scores were computed between AortaExplorer’s lumen segmentation and the AortaSeg24 ground truth, considering only labels corresponding to the aorta. Due to the difference in the definition of segments, the Dice score should not be interpreted as segmentation quality, but as structural overlap. Across 99 cases, the average Dice score was 0.84 (range: 0.61–0.93). For comparison, TotalSegmentator achieved an average Dice score of 0.87 (range: 0.75–0.92) against the same ground truth. The slightly lower Dice scores for AortaExplorer are explained by the inclusion of aneurysmal sacs with coagulated blood in the ground truth, which AortaExplorer intentionally excludes. In the reference study for AortaSeg24 [17], the highest reported average Dice score was 0.782 for the entire aortic tree. Although AortaExplorer focuses on the lumen rather than the full aortic structure, its performance remains highly competitive.

#### Aortic vessel tree (AVT) CTA dataset

**Fig. 18:**
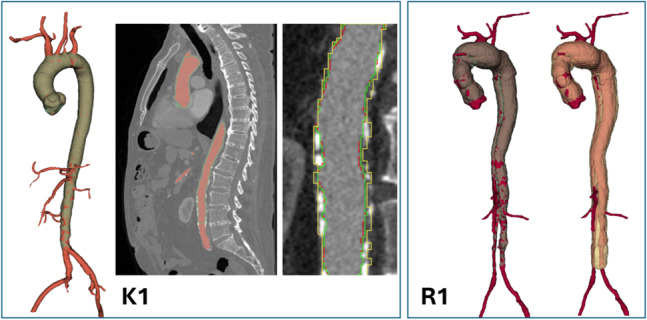
Results on the AVT dataset for two cases (K1 and R1). *K1:*) The ground truth segmentation is in red, the AortaExplorer is in green (Dice score = 0.88) and TotalSegmentator in yellow (Dice score = 0.83) (only on one cross section). *R1:*) The ground truth segmentation is in red, the AortaExplorer (Dice score = 0.89) is in green, and TotalSegmentator (Dice score = 0.81) in yellow

AortaExplorer successfully processed 49 of 56 scans; failures were due to absent contrast, image corruption, or incomplete initial segmentation by TotalSegmentator. A comparison of the segmentation results of AortaExplorer and TotalSegmentator compared to the provided ground truth can be seen in Fig. 18 for two cases. In case K1, AortaExplorer accurately segments the lumen and closely matches the ground truth, except where branch vessels are included in the ground truth mask (Dice score = 0.88). Both AortaExplorer and the ground truth exclude calcified deposits, whereas TotalSegmentator includes them. Case R1 involves an abdominal aortic dissection (Fig. 3); AortaExplorer and the ground truth agree (Dice score: 0.89), while TotalSegmentator includes the aneurysmal sac (Dice score = 0.81). Across all 49 successful cases, AortaExplorer achieved an average Dice score of 0.87 (range: 0.71–0.95), compared to 0.83 (range: 0.71–0.90) for TotalSegmentator. As with the AortaSeg24 results, the Dice score should not be interpreted as segmentation quality, but as structural overlap.

#### CIS-Unet dataset

AortaExplorer successfully analyzed all 59 scans. Compared to ground truth annotations, AortaExplorer achieved an average Dice score of 0.85 (range: 0.65–0.94), while TotalSegmentator reached 0.88 (range: 0.80–0.93). Differences are explained by the inclusion of aneurysmal sacs in the ground truth, which AortaExplorer intentionally excludes to focus on the contrast-enhanced lumen.

## Discussion

In this study, we introduce AortaExplorer, a comprehensive tool designed for end-to-end analysis of the aorta from CTA scans. AortaExplorer facilitates the extraction of image-based biomarkers and visualization of analytical output by integrating state-of-the-art methods, such as TotalSegmentator, as the basis for our aorta analysis. Although TotalSegmentator provides excellent initial segmentations, our evaluations revealed that refinement of these segmentations is crucial. Refinement helps with an accurate estimate of the aortic diameters by removing calcifications and the aortic wall from the segmentations. Furthermore, incorporating information about the surrounding organs allows us to accurately estimate the annulus landmark, which is not captured in the raw TS segmentation. The refinement results in large improvements in the mean absolute error between manual and AortaExplorer measurements: the smallest improvement is observed for the infrarenal diameter with a decrease of 43%, while the largest improvement is observed for the sinus of Valsalva diameter with a 70% reduction in mean absolute error.

AortaExplorer has undergone extensive validation using more than 10,000 CTA scans from the CGPS cohort. AortaExplorer extracts five main landmarks, dividing the aorta into its anatomical segments, and three additional landmarks at the aortic root. Six of these segments and landmarks correspond to manual measurements: annulus, sinus of Valsalva, sinotubular junction, ascending, descending, and infrarenal diameters. The tool demonstrated high and robust performance in estimating all six diameters in the large-scale dataset. AortaExplorer was evaluated on three publicly available aortic CTA datasets and demonstrated performance comparable to state-of-the-art methods in terms of Dice similarity scores. In addition to segmentation accuracy, AortaExplorer provides comprehensive visualization tools and quantitative measurements, enabling detailed anatomical assessment beyond what is typically offered by existing approaches.

In certain scenarios, validating automated measurements against manual readings is impractical due to time constraints or the complexity of specific measurements. In such cases, visualization becomes critical for quality assurance. Based on the visualization from AortaExplorer, as illustrated in Fig. 5, an experienced medical reader can quickly assess whether segmentation and landmark estimation were successful and if the extracted measurements can be trusted. Since a complete manual aortic analysis takes around 15 minutes, this method can drastically reduce the reading time, while maintaining high quality through human expert control. This would also be necessary to identify outliers or extreme estimation errors.

Although existing studies provide reference values for aortic tortuosity based on analyzes of limited patient groups (200 and 182 patients, respectively [1, 8]), and manual assessment for normal aortic diameter ranges (902 CT scans [29]), our current study advances this work by evaluating both full aortic and descending aortic tortuosity on 1,144 scans along with diameter measurements on 10,000 scans. This demonstrates the ability of tools like AortaExplorer to accelerate manual assessment tasks and allow the extraction of advanced geometric biomarkers in large cohort studies.

The 2024 ESC Guidelines for the management of peripheral arterial and aortic diseases [4] highlight the need for novel individualized risk stratification parameters beyond traditional markers for aortic aneurysms. AortaExplorer addresses this gap by enabling researchers to explore novel image-based biomarkers through possible custom-build functionalities built on top of our validated aorta segmentation and landmark detection. As an open-source tool, AortaExplorer has potential for extensive clinical research applications. It facilitates large-scale analysis of established image-based aortic biomarkers, enabling clinical researchers to establish reference values for these biomarkers on a large cohort scale. Furthermore, the adaptability of the tool enables exploratory research for the discovery of new image-based biomarkers.

Calcifications, identifiable due to their high X-ray attenuation and HU values, pose a challenge in contrast-enhanced scans where patient-specific HU values in contrast-enhanced blood complicate identification. An extensive robustness analysis was performed on the HU threshold parameters, and it was found that the default parameters provide a robust estimate of the ratio of calcification. Future research will focus on the possibility of deriving a ground truth calcification measure that can be used to further develop and validate calcification detection from AortaExplorer.

## Conclusion

In conclusion, this study presents AortaExplorer as a comprehensive tool for automated aortic analysis from CTA scans. By incorporating advanced segmentation refinement techniques, AortaExplorer significantly improves the accuracy of aortic diameter estimations from the TotalSegmentator baseline segmentations. Validated through extensive evaluation on a large-scale population data set, as well as smaller open source data sets, the tool demonstrates robust performance and offers clinical researchers the ability to conduct detailed cohort studies and explore novel biomarkers with efficiency and precision. Furthermore, AortaExplorer is open-source, making it a great resource for research on established and exploratory aorta biomarkers.

## Footnotes

1 https://github.com/RasmusRPaulsen/AortaExplorer

2 https://github.com/RasmusRPaulsen/AortaExplorer

## Declarations

### Code availability

The code for AortaExplorer is available at https://github.com/RasmusRPaulsen/AortaExplorer/.
